# Chemical and Morphological Characterization of Magnetron Sputtered at Different Bias Voltages Cr-Al-C Coatings

**DOI:** 10.3390/ma10020156

**Published:** 2017-02-10

**Authors:** Aleksei Obrosov, Roman Gulyaev, Andrzej Zak, Markus Ratzke, Muhammad Naveed, Wlodzimierz Dudzinski, Sabine Weiß

**Affiliations:** 1 Chair of Physical Metallurgy and Materials Technology, Brandenburg Technical University, Cottbus 03046, Germany; muhammad.naveed@b-tu.de (M.N.); sabine.weiss@b-tu.de (S.W.); 2 Boreskov Institute of Catalysis SB RAS, Novosibirsk 630090, Russia; gulyaev@catalysis.ru; 3 Department of Material Science, Welding and Strength of Material, Faculty of Mechanical Engineering, Wroclaw University of Science and Technology, Wrocław 50370, Poland; andrzej.zak@pwr.edu.pl (A.Z.); wlodzimierz.dudzinski@pwr.edu.pl (W.D.); 4 Chair of Experimental Physics II/Materials Science, Brandenburg Technical University, Cottbus 03046, Germany; markus.ratzke@b-tu.de

**Keywords:** XPS, AFM, MAX phase, TEM, chemical bonding, surface morphology

## Abstract

MAX phases (M = transition metal, A = A-group element, and X = C/N) are of special interest because they possess a unique combination of the advantages of both metals and ceramics. Most attention is attracted to the ternary carbide Cr_2_AlC because of its excellent high-temperature oxidation, as well as hot corrosion resistance. Despite lots of publications, up to now the influence of bias voltage on the chemical bonding structure, surface morphology, and mechanical properties of the film is still not well understood. In the current study, Cr-Al-C films were deposited on silicon wafers (100) and Inconel 718 super alloy by dc magnetron sputtering with different substrate bias voltages and investigated using Scanning Electron Microscopy (SEM), X-ray Photoelectron Spectroscopy (XPS), X-ray Diffraction (XRD), Atomic Force Microscopy (AFM), and nanoindentation. Transmission Electron Microscopy (TEM) was used to analyze the correlation between the growth of the films and the coating microstructure. The XPS results confirm the presence of Cr_2_AlC MAX phase due to a negative shift of 0.6–0.9 eV of the Al2p to pure aluminum carbide peak. The XRD results reveal the presence of Cr_2_AlC MAX Phase and carbide phases, as well as intermetallic AlCr_2_. The film thickness decreases from 8.95 to 6.98 µm with increasing bias voltage. The coatings deposited at 90 V exhibit the lowest roughness (33 nm) and granular size (76 nm) combined with the highest hardness (15.9 GPa). The ratio of Al carbide to carbide-like carbon state changes from 0.12 to 0.22 and correlates with the mechanical properties of the coatings. TEM confirms the columnar structure, with a nanocrystalline substructure, of the films.

## 1. Introduction

MAX phases are ternary carbides and/or nitrides with the composition form M*_n_*_+ 1_AX*_n_*, where *n* is 1, 2 or 3; M is an early transition metal; A is an A-group element from the periodic table of elements; and X is carbon or nitrogen [1]. Since their scientific discovery in the 1960s by Jeitschko et al. [2,3], MAX phases have gained significant attention due to their combination of the merits of metals and ceramics [4,5]. They exhibit high stiffness [6,7], easy machinability [5], self-healing behavior [8], chemical- [9,10], oxidation- [11,12] and hot corrosion resistance [13,14].

MAX-phase films have been synthesized using different chemical (CVD) and physical (PVD) methods. In 2002, Palmquist et al. [15] deposited Ti_3_SiC_2_ onto single-crystal substrates by Physical Vapour Deposition (PVD). Since then different PVD methods have been used for the deposition of MAX phases, including magnetron sputtering.

Nowadays, the ternary carbide Cr_2_AlC has a great potential for industrial application due to excellent high-temperature oxidation resistance and hot corrosion resistance due to the formation of a protective Al_2_O_3_ layer [16,17,18,19]. Cr_2_AlC film has been successfully deposited onto silicon wafers [20], single crystalline oxide [21,22], steel [23], Ni-based superalloy [17,24], and Ti alloy [25]. Cr_2_AlC has been produced using magnetron sputtering, either by sputtering from elemental targets [26] or compound targets [23]. Field et al. [27] deposited Cr_2_AlC coatings using direct current magnetron sputtering (DCMS) and high power impulse magnetron sputtering (HiPIMS).

It is well known that the microstructure and properties of PVD coatings strongly depend on deposition parameters such as deposition temperature, sputtering power, substrate bias, etc. [28]. The deposition temperature needed for the formation of Cr_2_AlC thin films was discussed in different works [13,23,24]. Walter et al. [23] reported the formation of Cr_2_AlC from a compound target at a substrate temperature of 450 °C. Li et al. [24] produced Cr_2_AlC coatings by magnetron sputtering from a bulk Cr_2_AlC target at a substrate temperature range of 370–500 °C. However, Abdulkadhim et al. [13] reported that a higher temperature is required for Cr_2_AlC formation. In a previous publication by Naveed et al. [17], the influence of sputtering power on growth and mechanical properties of Cr_2_AlC was already discussed. It was found that preferential coating growth orientation changes and a slight increase in hardness occurs with increasing sputtering power.

However, despite many studies on magnetron sputtered Cr_2_AlC films [13,17,23,24,26,27,29,30,31], up to now the effect of bias voltage on the chemical bonding structure, surface morphology, and mechanical properties of film is still not well understood.

The purpose of this work is to study the influence of bias voltage on the properties of Cr-Al-C coatings deposited onto IN718 and silicon wafers (100). The influence of the substrate bias voltage on the coating characteristics, such as chemical bonding structure, surface morphology, and roughness, was investigated using X-ray photoelectron spectroscopy (XPS) and atomic force microscopy (AFM). Mechanical properties were examined by means of nanoindentation. Transmission electron microscopy was used to analyze the correlation between the growth of the films and the coating microstructure.

## 2. Experimental Section

Cr-Al-C coatings were deposited on (100) silicon wafers and Inconel 718 super alloy (NiCr19Fe19Nb5Mo3), finding its application in gas turbines [32]. Inconel 718 specimens were polished using SiC paper up to 2400 grade, further cleaned ultrasonically, and dried in air. The coating deposition was performed in a CC800/9 industrial coater from CemeCon AG (Würselen, Germany). Prior to deposition, the specimens were etched under a vacuum at a pressure of 350 MPa for 30 min. Direct current magnetron sputtering was used with a compound target (88 × 500 mm^2^) with a Cr:Al:C composition of 2:1:1 supplied by CemeCon AG. The sputtering process was performed in static mode. The deposition temperature was 550 °C because this temperature is shown in the literature as the lowest temperature, where Cr_2_AlC formation is ensured [13]. The distance between the target and the substrate was 70 mm. Further details of the deposition process parameters for Cr-Al-C coatings are given in Table 1.

A scanning electron microscope (SEM, TESCAN Mira, Prague, Czech Republic) was used to investigate the growth of the films on (100) Si wafers. Crystallographic phases and XRD patterns of the films were identified using a D8 Discover X-ray diffractometer (Bruker AXS, Karlsruhe, Germany) with a grazing incidence X-ray diffraction (GIXRD) method at a low incident angle of 9° with a Cu Kα source (λ = 0.15406 nm) operating at 40 kV and 40 mA.

An ES300 KRATOS spectrometer (Kratos Analytical, Manchester, UK), equipped with an X-ray tube with twin Mg and Al anodes, was used for the XPS studies. Prior to the installation of the samples into the spectrometer, their surfaces were cleaned by washing them several times in pure ethanol. Photoelectron spectra were acquired after evacuation to an ultra-high vacuum (UHV, 5 × 10^−9^ mbar). The electron analyzer was calibrated against Au4f7/2 (84.0 eV) and Cu2p2/3 (932.7 eV) photoelectron line positions obtained from Ar^+^ ion cleaned metallic surfaces of copper and gold. Non-monochromated Mg Kα-radiation was used for sample analysis. Due to the conductivity of the samples, the XP-spectra were presented without calibration.

The spectra processing was performed using XPS-Calc software (Boreskov Institute of catalysis, Novosibirsk, Russia) [33]. Doniach-Sunjic asymmetric lineshape was used for the description of the components related to carbide phase in Cr2p, Al2p, and C1s spectra due to the conductive character of this phase. The sample coated at 90 V bias voltage was characterized by XPS after evacuation to UHV and after Ar^+^ ion etching at 1 keV and 20 mA for 30, 60, 90 and 120 min. The samples prepared with other bias voltages were characterized after 120 min ion etching.

The surface morphology of the films was analyzed by AFM (“Smena” microscope by NT-MDT, Zelenograd, Russia) operating in contact mode with cantilever NSG 11 Pt (NT-MDT). The AFM observation was supported by TEM methods, using a Hitachi H-800 transmission electron microscope (Hitachi High-Technologies Corp., Tokyo, Japan) with an accelerating voltage of 150 kV. Cross-sectional samples were prepared using the focused ion beam (FIB) technique. The selected area diffraction patterns were analyzed using software developed in the Electron Microscopy Lab at Wroclaw University of Technology. In order to determine the mechanical properties (hardness and E-Modulus) of the coatings, nanoindentation tests were carried out using a UNAT nanoindenter (ASMEC GmbH, Radeberg, Germany) with a Berkovich Indenter (three-face pyramid diamond) by means of the Quasi Continuous Stiffness Method (QCSM). A load of 15 mN was used, hence the penetration depth did not exceed 10% of the total coating thickness in order to neglect the substrate effect [17,32]. The load-displacement curves were analyzed using the Oliver Pharr method [34]. For each sample, the mean value of twenty single measurements was calculated.

## 3. Results and Discussion

### 3.1. Scanning Electron Microscopy

**Figure 1:**
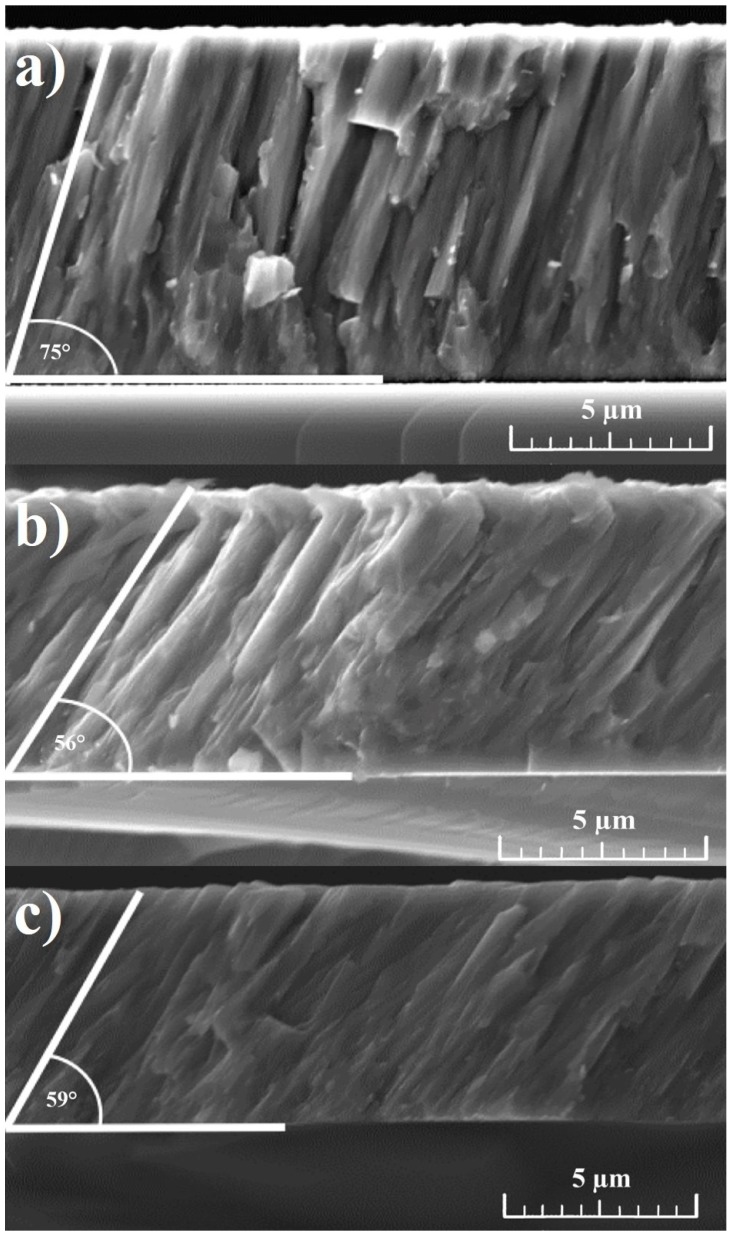
Cross-section SEM images of the Cr-Al-C coatings deposited at various bias voltages (**a**) 60 V; (**b**) 90 V; and (**c**) 120 V.

The coating growth was analyzed by means of SEM, as shown in Figure 1. The cross-sectional view of the coating deposited at 60 V shows a collection of dense columns grown on the substrate with an angle of nearly 75° with respect to the substrate. Surface analysis indicates a feathery structure showing apparent columns of various diameters. According to the Thornton model, this structure could be attributed to Zone-T [35]. With the increase of substrate voltage, a clear change in column growth can be seen. Different growth angles of approximately 56° and 59° can be observed for Cr-Al-C during growth at 90 V and 120 V, respectively. Naveed et al. [17] observed similar columnar structures with certain column orientations relating to the substrate at sputtering powers up to 2.5 kW.

### 3.2. XRD

**Figure 2:**
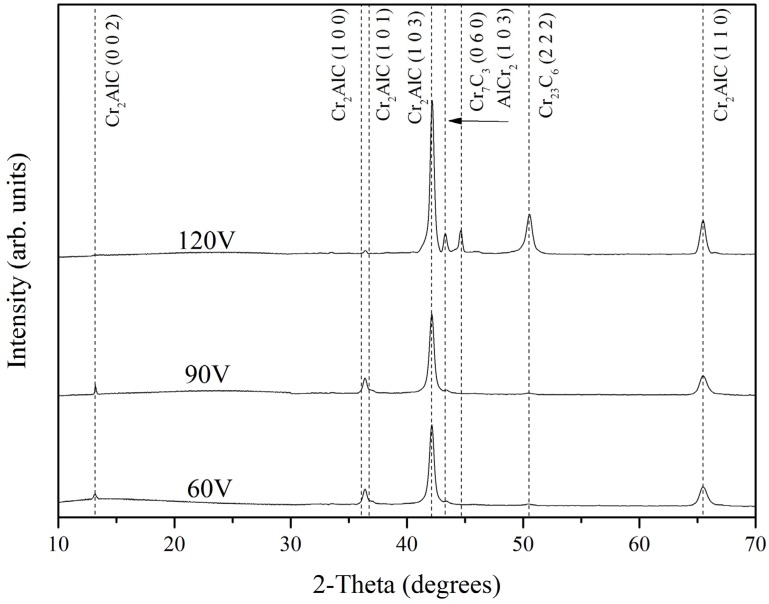
X-ray diffraction (XRD) patterns of the deposited Cr-Al-C coatings.

Representative X-ray diffraction (XRD) patterns of the deposited Cr-Al-C coatings obtained by GIXRD are given in Figure 2. In the range 10°–70°, there are two super lattice reflections, (002) and (101), corresponding to the ordered Cr_2_AlC MAX phase [29,36]. At around 13.3°, (002) weak reflection for coatings at 60 and 90 V was found, identifying the presence of the MAX phase [29]. The XRD patterns corresponding to 60 V and 90 V are very similar. These two coatings show the Cr_2_AlC (103) as the dominant orientation and a small amount of (100/101) of the same phase. The XRD pattern of 120 V is different to the others. The Cr_2_AlC (103) peak is stronger for 120 V, which can be related to larger grains, higher crystallinity, or more MAX phase. This is in good correlation with the results of Field et al. [27], who showed for DCMS and HiPIMS deposited coatings that Cr_2_AlC (103) reflection was the highest. Furthermore, the coating deposited at 120 V displays the presence of Cr_7_C_3_, AlCr_2_, and Cr_23_C_6_ peaks.

### 3.3. X-ray Photoelectron Spectroscopy

**Figure 3:**
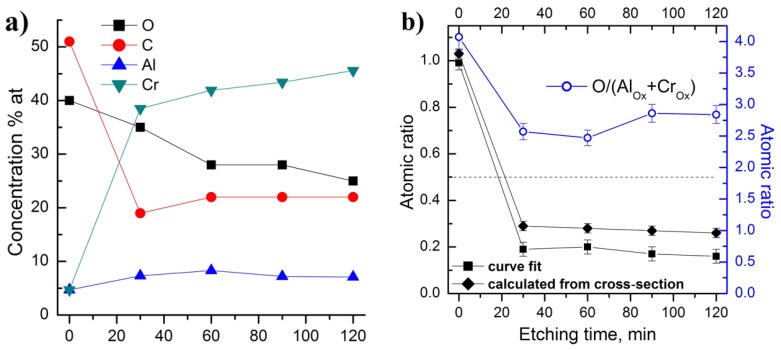
(**a**) Atomic composition at the surface; (**b**) O/(Al_Ox_ + Cr_Ox_) and Al/Cr atomic ratios for Cr-Al-C coating at 90 V as a function of Ar^+^ etching time obtained two different ways.

XPS analysis allows information to be obtained about the chemical structure of the elements on the surface of samples. The samples were exposed to air prior to the XPS measurements. Therefore, the initial surface of all samples was oxidized and strongly contaminated. Survey spectra revealed Cr, Al, C and O peaks, and a carbon content of more than 50% was measured on the surface (Figure 3a). Cr as well as Al concentrations on the untreated surface were observed at about 5%. The coating deposited at 90 V was measured after 30, 60, 90 and 120 min of argon ion etching (Ar^+^ etching rate: 2 Å/min) in order to study the chemical composition of the surface layers. Figure 3b shows the atomic concentration of the elements versus etching time.

Argon etching led to a decrease of C and O and to an increase of Cr and Al concentration due to the removal of contaminated and oxidized layers on the surface. The composition reached a constant value after 60–90 min of argon etching. It is noticed that after 120 min of etching, oxygen still remained at approximately 20%. It is assumed that this amount of oxygen was incorporated into the coatings during deposition. The analysis of the deposition process reveals that the most likely source of oxygen contamination may be residual oxygen in the deposition chamber. The oxygen incorporation in the structure of M_2_AlC phases is well known [31,37]; however in our case the concentration is higher than expected.

**Figure 4:**
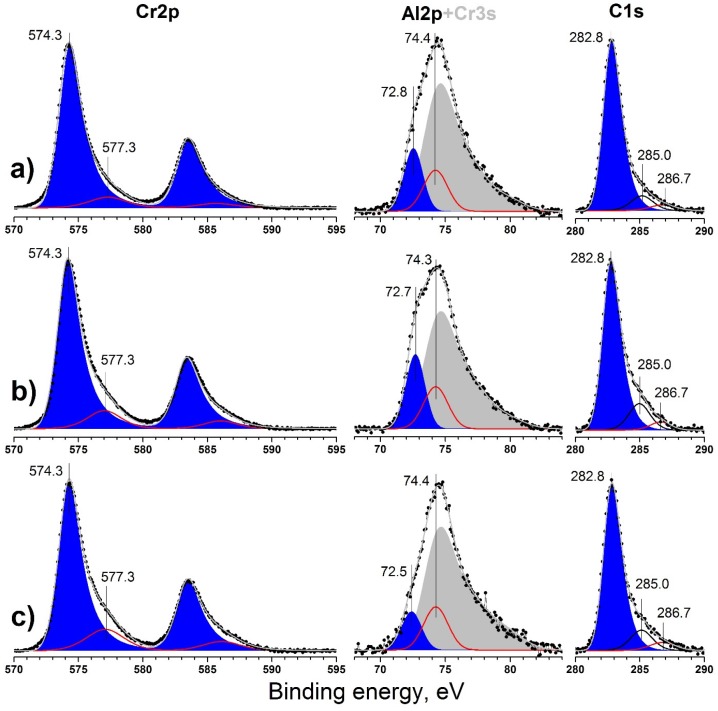
Cr2p, Al2p, and C1s XP-spectra obtained for Cr-Al-C films at (**a**) 120 V; (**b**) 90 V; and (**c**) 60 V after Ar^+^ ion etching for 120 min.

Note that the initial surface was depleted with chromium; the Al/Cr ratio was about 1. The chromium concentration sharply increased in the first 30 min of etching and slightly changed during further etching, while the aluminum concentration remained almost constant. Thus, the inner layers (6–24 nm depth) were depleted with aluminum. The upper limit of the Al/Cr ratio (Figure 3b) was estimated to be less than 0.35, which was substantially lower than the specified value of 0.5. The origin of such a low Al/Cr ratio obtained from XPS is discussed below (see the description of Al2p + Cr3s lines presented on Figure 4). Thus, all samples were substantially depleted with aluminum in the subsurface layers. The amount of oxygen on the cleaned surfaces increased with the decrease in substrate bias from 120 to 60 V.

Typical high resolution XPS spectra of Cr2p, Al2p, and C1s core levels of coatings prepared at different bias voltages are presented on Figure 4a–c. Etching of the surface for 120 min led to almost complete removal of oxidized Cr^3+^ species and carbon contaminants.

The carbon valence state represented mainly by the peak at 282.8 is typical for carbide-like carbon in all cases, i.e., typical for the Cr–C bond [38]. Higher than typical values for Al_4_C_3_ (282.2 eV) indicate the absence of a discrete aluminum carbide phase [39]. Only minor additional components were observed at 285.0 and 286.6 eV, which corresponds to elementary carbon (C–C bonds) and oxidized carbon species (C–O). They were probably formed due to the decomposition of carbide phases into pure elements under ion beam influence (Ar ion damage [40]) and the subsequent removal of the decomposition products [41,42]. Thus, a steady-state concentration of elementary carbon species was observed.

Chromium was represented by two states with binding energies; (*BE*) = 574.3 and 577.3 eV. Minor components at 577.3 eV corresponding to Cr_2_O_3_ were observed [43]. The main asymmetric component at 574.3 eV corresponds to chromium in carbide phase [44] or in metallic Cr [38]. Similar XPS results were presented by Zamulaeva et al. for Cr_2_AlC prepared using pulsed electrospark deposition (PED) [45]. Furthermore, both peaks are situated in the same position for all coatings, and no chemical shift could be discerned.

The minor Cr3s photoelectron line overlaps with the Al2p main photoelectron line of aluminum. The estimation of Al2p contribution to this spectrum is possible via two ways. Theoretical Al2p contribution can be estimated using the Cr2p photoelectron spectra and Scofield cross-sections [46] of the 3s and 2p core-levels of chromium for the calculation of the Cr3s line intensity. Thus, the theoretical Al/Cr ratio could be obtained. The most probable experimental Al/Cr ratio can be estimated via the curve of best fit of the experimental spectrum from the total areas of the peaks at 72.7 and 74.4 eV in the Al2p + Cr3s on Figure 4 using binding energy and the shape of Cr3s taken from [43,47,48]. The Al/Cr ratios obtained each way are presented in Figure 3b. The Al/Cr ratio was calculated to be about 0.3 using the theoretical approach and about 0.2 using curve fitting of the experimental spectra, which were both lower than the specified value 0.5.

**Figure 5:**
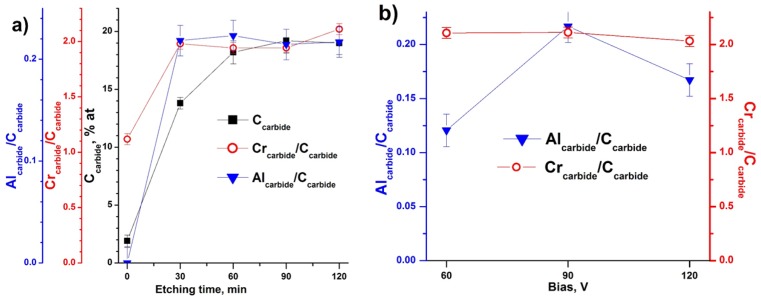
(**a**) Atomic concentration of carbide-like carbon in relation to all elements; atomic ratio of chromium carbide (574.3 eV) and aluminum carbide (72.5–72.8 eV) states to carbide like carbon (282.8 eV); and Ar^+^ etching time obtained for Cr-Al-C at 90 V; (**b**) Cr_carbide_/C_carbide_ and Al_carbide_/C_carbide_ ratios obtained for all samples after 120 min of Ar^+^ ion etching vs. bias voltage.

Aluminum was much more oxidized compared to chromium due to its more metallic chemical properties, as obvious from the intensity of the peak at 74.3–74.4 corresponding to Al_2_O_3_ [49,50]. The accuracy of the Al2p + Cr3s photoelectron line fitting is confirmed by the quotient of the total oxygen amount and the oxidized Al + Cr species (doublet peak at 577.3 eV in Cr2p and peak at 74.4 in Al2p + Cr3s), which is close to 3 and presented in Figure 5b. Thus, these peaks in the Cr2p and Al2p + Cr3s lines correspond to Cr_2_O_3_ and Al_2_O_3_, respectively.

The peak at 72.8–72.5 eV formally corresponds to metallic aluminum, which has a binding energy of about 72.6–72.8 eV [39,51]. Aluminum carbide is characterized by a higher binding energy of Al2p core level 73.4 eV [39]. Thus, taking into account the rather high reactivity of aluminum towards carbon [39], we attribute this state to Cr_2_AlC. The negative shift 0.6–0.9 eV relative to pure aluminum carbide can be explained with more metallic Al–C bonds in Cr_2_AlC phase compared to Al_4_C_3_. Zhang et al. observed a similar negative shift of the Al2p core level for Ti_2_AlN phase compared to pure AlN. Thus, such a shift could be used as indicator of MAX phase formation [51,52]. In the current study, the XPS results could confirm the formation of a Cr_2_AlC MAX phase, and these results are in good agreement with the XRD measurements.

The Cr and Al carbide states standardized on carbide-like C reached a constant value after 30 min of ion etching, while total carbide-like C concentration reached a constant value only after 60 min of ion etching (Figure 5a). Thus, despite the removal of carbon contaminants and oxidized species, the carbide phase composition is constant in the topmost ~24 nm; however, this phase is deplete of aluminum and corresponds to Cr2:Al0.25:C1 stoichiometry in the case of the Cr-Al-C coating at 90 V (Figure 5a). Taking into account the oxidation of the surfaces with oxygen traces during ion etching, the initial “non-oxidized” surface composition was estimated to correspond to the Cr2:Al0.6–0.7:C1 stoichiometry under this assumption.

Moreover, the Cr carbide to carbide-like C state ratio was very stable, independent of the bias voltage (Figure 5b), and equal to 2.03–2.1, while the Al non-oxidized carbide state to carbide-like C state varied from 0.12 to 0.22.

### 3.4. Atomic Force Microscopy

AFM gives information about the morphological properties of films. The main characteristics of Cr-Al-C coatings like thickness, surface roughness, and granular size are listed in Table 2. Thickness, as a function of bias voltage, shows an inverse relationship with the bias. The film thickness reduces from 8.95 to 6.98 µm, which can be related to the notable sputtering off of the film (re-sputtering) [53,54].

During increasing bias voltage, the incident ion energy rises and, therefore, more atoms from the growing coatings will be re-sputtered [55,56]. These results are in good correlation with the literature [53,57,58,59]. However, Jiang et al. showed that, at a low bias voltage (till 50 V), the thickness can increase with rising bias until the ion current density is saturated [54].

**Figure 6:**
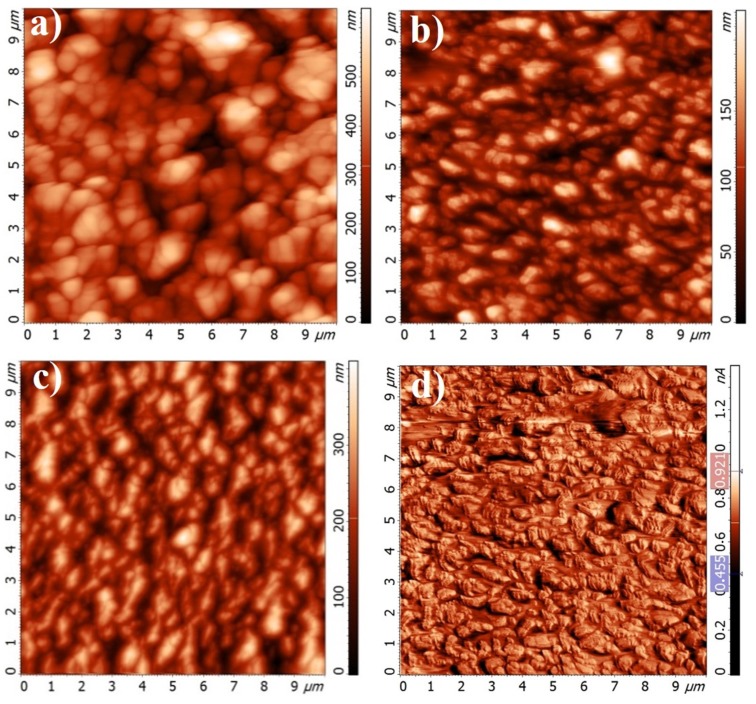
Atomic force microscopy (AFM) topography images of Cr-Al-C films on a Si (100) wafer at (**a**) 60 V; (**b**) 90 V; (**c**) and 120 V; (**d**) A lateral force map at 90 V.

The surface morphologies of the coated silicon substrates were examined by the AFM method and shown in Figure 6. All films have a granular structure with visible agglomerated grains. The Cr-Al-C coating deposited at 60 V exhibits the morphology with the highest RMS (root-mean-square). As the substrate bias changes from 60 to 90 V, the structure changes from irregular to rectangular particulates with random alignment and the roughness sharply decreases to 33 nm, indicating a surface-smoothing phenomenon.

The lateral force map for the sample deposited at 90 V with the pronounced superstructure of the films is presented in Figure 6d. A step-line structure is evident, and its edges are visible. At 120 V, the film became again rougher with a roughness of about 60 nm (Figure 6c). The increase in roughness with rising bias voltage can be explained by higher ion energy [60,61].

The average granular size was obtained using atomic force microscopy. Similar to the roughness, the granular size significantly decreases as the bias increases from 60 V to 90 V and then rises during a further increase to 120 V.

Reduction of granular size can also be related to a modification of the superficial morphology by argon ion bombardment, increasing the energy associated with the atoms on the substrate surface and/or the growing coating surface [62]. In addition, Lee et al. [63] reported that applied DC bias may cause an increase in the point defects in the film’s structure, increasing compressive residual stress and reducing the grain size. However, as mentioned above, when the bias voltage increases, the ion energy increases, resulting in higher adatom mobility. High energy promotes diffusion as well as grain boundary migration that might cause increasing granular size [60,64]. Similar results were also reported by Gangopadhyay et al. [53] and Zhang et al. [65]. As available from Table 2, the first increase in bias voltage results in a decreasing granular size, while a further increase in bias voltage leads to an increase in granular size. Therefore, in the literature, both effects were discussed contradictorily. Thus, a superposition of different effects takes place. The problem of whether higher adatom mobility, higher ion energy, and more defects result in smaller or larger granular size has still not been solved [53,65].

### 3.5. Transmission Electron Microscopy

**Figure 7:**
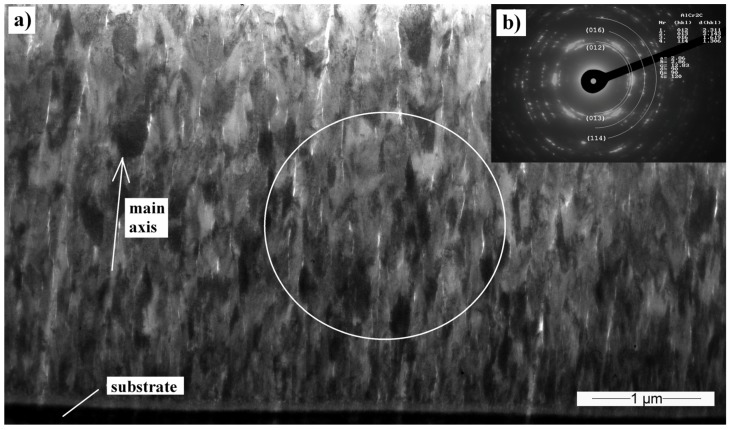
(**a**) Transmission electron microscopy (TEM) cross-sectional bright-field (BF) image of the Cr_2_AlC coating, deposited at 90 V; (**b**) Selective area electron diffraction (SAED) obtained in the place marked by the circle.

Cross-sectional TEM samples of the Cr_2_AlC film deposited at 90 V were analyzed by means of TEM to understand the coating growth. The coating layer exhibits a columnar polycrystalline growth structure consisting of 120–250 nm width columns (Figure 7a). The selective area electron diffraction taken from a 3-µm diameter circle shows a textured coating with preferential columnar orientation. Cr_2_AlC phase was recognized and the Cr_2_AlC planes (012) (*d*_012_ = 0, 2311 nm), (013) (*d*_013_ = 0, 2143 nm), (016) (*d*_016_ = 0, 1619 nm), and (008) (*d*_008_ = 0, 1346 nm) were detected. The coating is visibly textured. The angle measurement of the most varied diffraction spots in the same interplanar distance showed that most of the grains are oriented in ±15° angle to the main growth axis.

**Figure 8:**
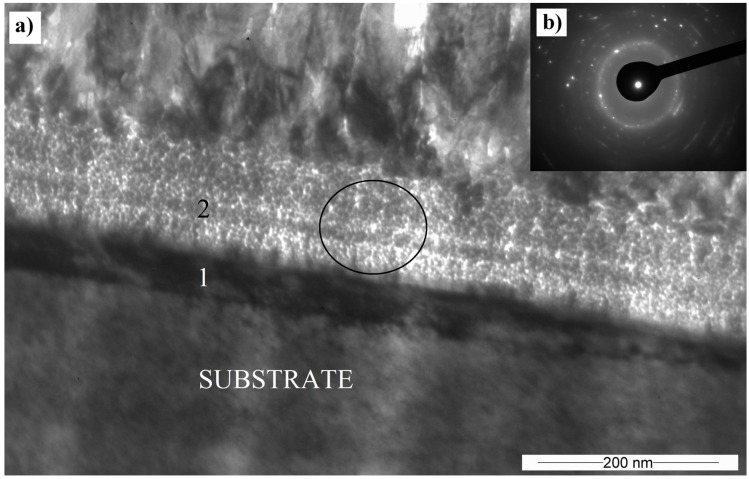
(**a**) TEM cross-sectional BF image of the Cr_2_AlC coating interface, deposited at 90 V (1; substrate part of interface, 2; coating part of interface); (**b**) Selective area electron diffraction (SAED) obtained in the place marked by the circle.

The substrate-coating interface region shows the presence of two layers marked with the numbers 1 and 2 in Figure 8. An amorphous or nanocrystalline layer marked with number 1 has about a 50–60 nm thickness. Between the amorphous region and the columnar crystal, a 100–120 nm nanocrystalline layer exists. In this region, the beam is strongly dispersed but has not created a circular polycrystalline pattern. It is possible to observe individual crystalline reflections, but the pattern has the attributes of an amorphous or nanocrystalline structure.

**Figure 9:**
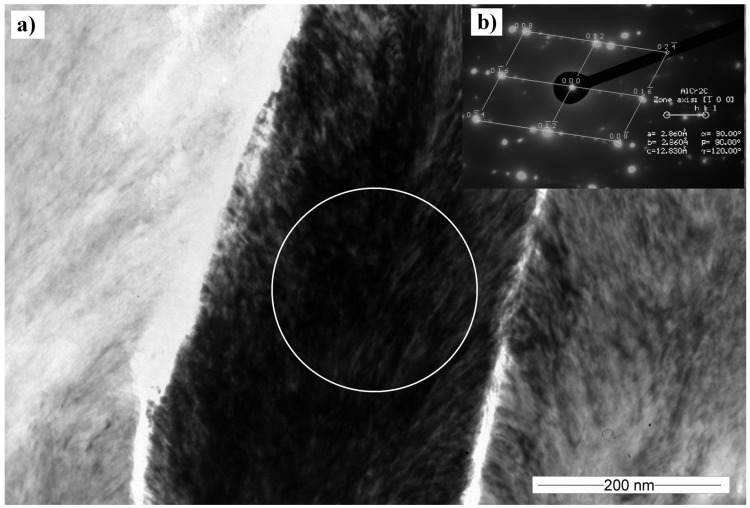
(**a**) TEM cross-sectional BF image of the single Cr_2_AlC crystal on the top layer of the coating, deposited at 90 V; (**b**) Selective area electron diffraction (SAED) obtained in the place marked by the circle.

The diffraction taken in a single grain confirms the presence of Cr_2_AlC phase (Figure 9a). The selected area electron diffraction reveals blurred fuzzy streaks and extra diffraction spots along the direction [016] (Figure 9b). They can be caused by the formation of a superlattice structure inside the Cr_2_AlC grain, ordered stacking faults, or another long-period ordered crystal arrangement. This theory corresponds to the bright-field image, showing an oriented substructure inside the Cr_2_AlC grain (Figure 9a). Further results of the TEM analysis of the crystal growth will be published in the near future.

### 3.6. Nanoindentation

The hardness (*H*), elastic modulus (*E*), and the *H*/*E* and *H*^3^/*E*^2^ ratios of the coatings were obtained using a nanoindenter. The results had shown that the mechanical properties of the coating were influenced by the bias values (Table 3). The hardness and E-modulus values rapidly increased from 8.8 to 15.8 GPa and from 223.4 to 307.7 GPa, respectively, when bias rose from 60 V to 90 V. By further increasing the bias voltage up to 120 V, *H* and *E* were decreased. The *H*/*E* and *H*^3^/*E*^2^ values possessed maximums of 0.042 and 0.052, obtained at a bias voltage of 90 V. High *H*/*E* and *H*^3^/*E*^2^ values mean larger elastic strain to failure and higher fracture toughness [66]. The high hardness at 90 V is probably due to the reduction in granular size caused by the ionic bombardment [62]. Consequently, this enhancement of the mechanical properties with the increase in the applied bias voltage is caused by a blockage for the displacement of the cracks, and thus the energy necessary for the motion of cracks through the coating increases [67]. As previously reported in the literature, an increase in substrate bias raises the level of compressive residual stresses due to an increasing number of impinging ions onto the substrate as well as higher ion current density [68,69]. The XPS results show a change in the ratio of Al carbide state to carbide-like C state, determining the mechanical properties of the films. This statement needs further investigation and more detailed analysis. The nanoindentation results are consistent with the previous work of the authors [17], with hardness values in the range between 11 and 14 GPa at various sputtering powers as well as with further investigations about Cr_2_AlC [6,25]. Schneider et al. [6] measured the hardness value of Cr_2_AlC as 13 ± 2 GPa. Zamulaeva et al. [25] reported a hardness of 15.4 GPa and an elastic modulus of 288 GPa for coatings produced using pulsed electrospark deposition (PED). These hardness values are rather low in comparison to the nanoindentation data of Ti_2_AlC [70] and Ti_3_AlC_2_ [71] and are similar to V_2_AlC coatings [72,73] prepared using magnetron sputtering. Furthermore, the hardness values presented above are significantly higher than those for bulk MAX phases, which are typically 2–4 GPa [1]. This increase could be explained by the Hall-Petch’s effect [74], wherein the strength of materials is inversely proportional to the square of the crystalline size before a decreased threshold value. Due to the rapid cooling during PVD vapor condensation, the coatings developed nanocrystalline structure, which can be attributed to the better mechanical properties of coatings, compared to bulk material, and confirmed for different MAX phase coatings [6,72,73].

## 4. Conclusions

The chemical bonding structure, surface morphology, roughness, and mechanical properties of Cr-Al-C thin films deposited at different substrate bias voltages by direct current magnetron sputtering were studied. X-ray photoelectron spectroscopy (XPS) results reveal an unusual negative shift of 0.6–0.9 eV of the Al2p peak relative to pure aluminum carbide. The predominantly metallic Al–C bonds can explain this shift in Cr_2_AlC phase, which can be used as indicator for Cr_2_AlC MAX phase formation. These results can be confirmed by means of XRD measurements. The film thickness reduces from 8.95 to 6.98 µm due to re-sputtering. Granular size and roughness were obtained using atomic force microscopy (AFM), with the applied substrate bias changing from 60 to 90 V the roughness decreases and subsequently increases due to the further rise of the substrate bias to 120 V. Transmission electron microscopy (TEM) reveals a columnar structure with a nanocrystalline substructure in all the films and confirms the granular size determined by means of AFM. Nanoindentation shows that hardness increases significantly with the increase of bias voltage from 60 to 90 V and then decreases for 120 V. This trend of hardness can be explained with a change in granular size as well as the level of compressive residual stresses due to ionic bombardment. XPS shows a change in the ratio of Al carbide to carbide-like C state (from 0.12 to 0.22), which affects significantly the mechanical properties of the coatings. Hence, more detailed investigations on this topic are planned for the near future.

## Figures and Tables

**Table 1:** Deposition parameters of Cr-Al-C coatings.

Parameter	Value
Ar Pressure	600 MPa
Cathode Power	2.5 KW
Deposition Temperature	560 °C
Bias Voltage	−60 V/−90 V/−120 V
Deposition Time	1 h

**Table 2:** Effect of the bias voltage on the morphological properties of the films.

Bias Voltage (V)	Thickness (µm)	RMS (nm)	Granular Size (nm)
60	8.95	100	179
90	7.16	33	76
120	6.98	60	134

**Table 3:** Effect of the bias voltage on mechanical properties of the coatings.

Bias (V)	Hardness (GPa)	E-Modulus (GPa)	*H*/*E*	*H*^3^/*E*^2^
60	8.8 ± 0.9	223 ± 20	0.014	0.039
90	15.9 ± 3.2	308 ± 73	0.042	0.052
120	10.3 ± 0.5	257 ± 14	0.017	0.040
